# Piloting a forced-choice task to elicit treatment preferences in geographic atrophy

**DOI:** 10.1186/s13104-023-06531-8

**Published:** 2023-09-30

**Authors:** Jamie Enoch, Arevik Ghulakhszian, Mandeep Sekhon, David P. Crabb, Deanna J. Taylor, Christiana Dinah

**Affiliations:** 1 Department of Optometry and Visual Sciences, City, University of London, London, UK; 2 Ophthalmology Department, London North West University Healthcare NHS Trust, Central Middlesex Hospital, London, UK; 3 Centre for Applied Health and Social Care Research, Kingston and St George’s, University of London, London, UK; 4 Department of Brain Sciences, Imperial College, London, UK

**Keywords:** Geographic atrophy, Complement inhibitors, Intravitreal injections, Acceptability, Qualitative research

## Abstract

**Objective:**

Geographic Atrophy (GA) is the advanced form of the non-neovascular (‘dry’) type of age-related macular degeneration (AMD) and responsible for one-quarter of legal blindness in the UK. New therapies delivered by intravitreal injection are in late-stage development, and two such therapies (pegcetacoplan (Syfovre) and avacincaptad pegol (Izervay)) have now been approved for clinical use by the US Food and Drug Administration. These therapies slow down, but do not stop or reverse, progression of GA and they may also increase the risk of developing the neovascular (‘wet’) type of AMD. Within a larger study exploring the acceptability of these new treatments to people living with GA, we developed a forced-choice exercise to evaluate how participants weigh up benefits and drawbacks of different treatment regimens. This research note reports quantitative and qualitative findings from this exercise.

**Results:**

Twenty-eight participants took part in this exercise. The exercise demonstrated that participants were generally, although not unanimously, in favour of less frequent treatment for GA that was slightly less efficacious in terms of preserving visual function but presented a lower risk of developing wet AMD. Even among a small sample, the exercise demonstrated the highly personal and idiosyncratic decision-making processes influencing participants’ choices of preferred hypothetical GA treatment.

**Supplementary Information:**

The online version contains supplementary material available at 10.1186/s13104-023-06531-8.

## Introduction

Geographic Atrophy (GA) is the advanced form of the non-neovascular (‘dry’) type of age-related macular degeneration (AMD). GA accounts for approximately one-quarter of legal blindness in the UK [1] and globally, around 5 million people have GA in at least one eye [2], with the incidence expected to rise with ageing populations. About one-half of patients develop GA in both eyes within seven years of initial diagnosis [3]. In addition, up to 25% of eyes presenting with wet AMD may have concurrent GA at baseline [4]. There is currently no therapy for GA in the UK, a significant unmet need.

Positive results from phase 3 clinical trials of intravitreal complement inhibitors provide hope for a treatment for GA in clinical practice [5, 6]. Findings from the DERBY and OAKS trials of pegcetacoplan have shown that at 24 months, GA lesion growth was reduced by 21% with monthly intravitreal injections and 17% with every-other-month injections [7]. In the GATHER2 phase 3 trial of avacincaptad pegol, monthly intravitreal injections significantly reduced mean rate of GA growth over 12 months by 14.3% [6]. Indeed, in February 2023, the first-ever treatment for GA, pegcetacoplan, was approved for use by the Food and Drug Administration (FDA) in the US under the brand name Syfovre, based on reduced rates of lesion growth in the DERBY and OAKS trials [8]. In August 2023, avacincaptad pegol (brand name Izervay) was approved by the US FDA for clinical use, on the basis of reduced rates of GA growth in the GATHER1 and GATHER2 clinical trials [9, 10]. However, Syfovre and Izervay are yet to be approved in the UK (the location of the present study), and little is known regarding the hypothetical acceptability of these new treatments to patients.

In a previous study, our group explored the acceptability of these emerging new treatments for GA, using a questionnaire and qualitative analysis of interviews with open-ended questions [11]. With the help of a patient advisory group, composed of eight individuals with lived experience of GA who did not participate in this study but who generously volunteered their insights, we sought to understand the best way to communicate reduction in progression of GA lesions. Unanimously, our patient group felt that percentage reduction was difficult to understand as a measure of benefit and that expressing the benefit as additional time to participate in vision-related activities was optimal. As an extension of this exploratory mixed-methods study, we piloted use of a forced-choice task, with treatment scenarios based on the method of discrete choice experiments (DCE), to explore how participants weighed up the benefits and drawbacks of different potential treatment regimens. Our focus was less on the *quantitative* dimensions of which option was most commonly selected, and more on using these DCE-style scenarios as a tool to elicit *qualitative* data regarding participants’ preferences, concerns and uncertainties regarding the different hypothetical treatments.

## Methods

A more detailed account of our methods is provided in the published study protocol [12]. In brief, the forced-choice exercise using DCE-style scenarios reported in this research note was nested within a broader cross-sectional, mixed-methods design. Ethics Committee approval was obtained from the NHS Health Research Authority on 23 March 2021 (IRAS Project ID: 287824), and the study adhered to the tenets of the Declaration of Helsinki.

Thirty participants with a diagnosis of GA secondary to AMD were recruited from two Medical Retina clinics in London, UK. A maximum variation sampling approach was adopted, aiming to include a mix of individuals with previous experience of intravitreal injections for AMD and those naïve to injections, and those at varying stages of GA and in different living situations.

**Fig. 1:**
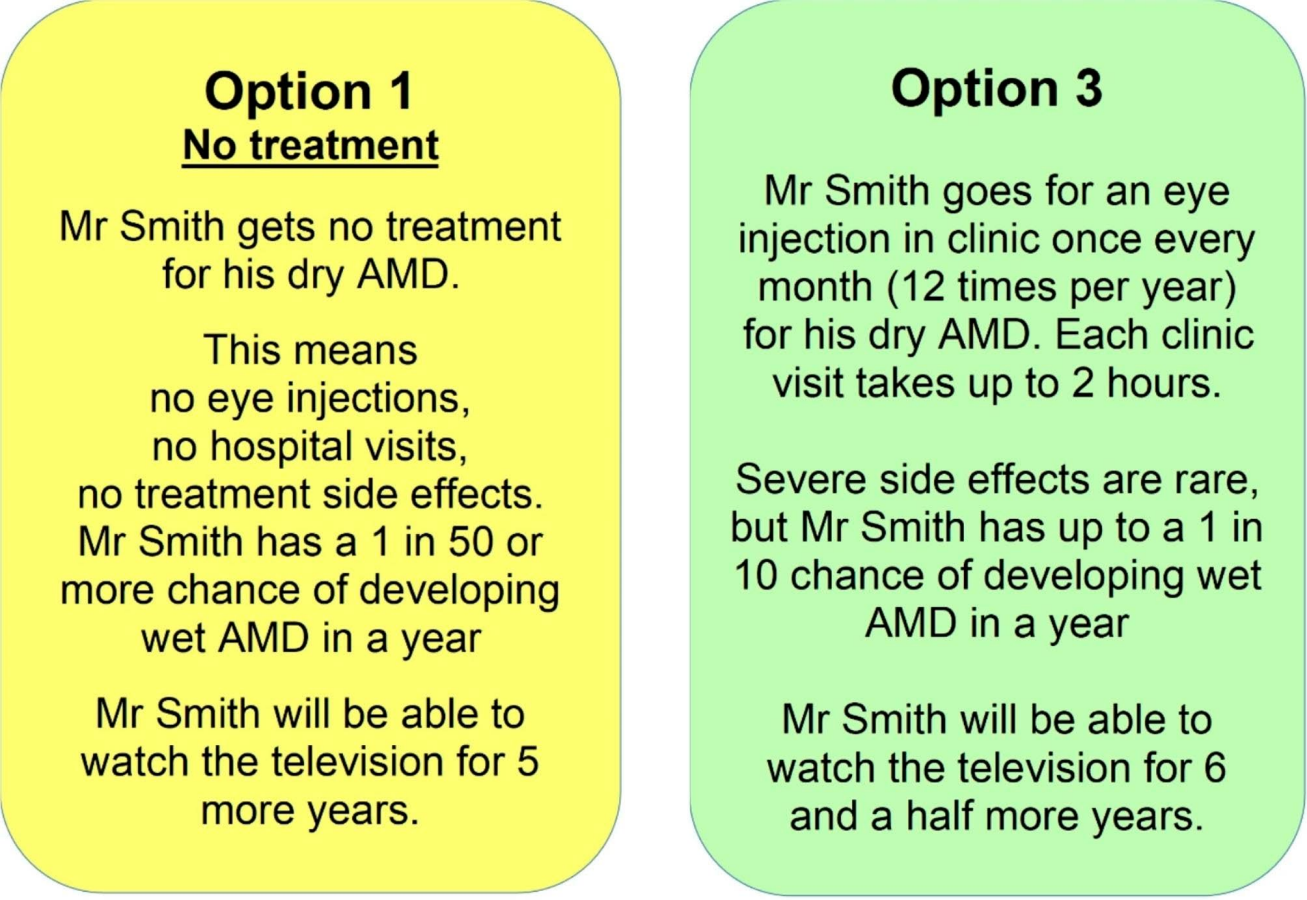
Example of two hypothetical treatment options for comparison

Following an interview in-person or by phone (conducted by authors AG, CD or JE), participants were invited to compare four different hypothetical treatment options for an imaginary patient. Option 1 was the baseline, ‘no treatment’ option, while the remaining three options were modelled on three candidate intravitreal treatments in pipeline development for GA and the available knowledge at the time: Pegcetacoplan [13], Avacincaptad pegol [14] and Brimonidine [15]. (Brimonidine is no longer in development as a treatment for GA). Participants were presented with cards showing the four different options, and were asked – in random order – to compare the six different possible pairs of options. The different attributes of interest (e.g. frequency of injection) and levels (e.g. once per month, once every-other-month) are displayed in Table 1. An example choice pair is shown in Fig. 1. At each stage, participants were asked to think out loud and explain the factors influencing their decision in as much detail as possible.

**Table 1:** Options presented in the forced-choice task, the different attributes and levels for each treatment option, and participants’ expressed preferences

Attribute	Option 1 (Baseline, No treatment)	Option 2	Option 3	Option 4
Injection frequency	0 injections	1 injection every other month (6 per year)	1 injection every month (12 per year)	1 injection every 3 months (4 per year)
Time spent in clinic for each injection	0 h	2 h	2 h	2 h
Risk of developing wet AMD within the next year	1 in 50	1 in 20	1 in 10	1 in 50
Functional vision preservation	5 years	6 years	6.5 years	5.5 years
**Number of participants expressing clear preference** (%)*	6 (21)	3 (11)	5 (18)	8 (29)

*Six participants (21%) expressed no clear preference for any single option

Attributes for the different treatment scenarios were selected on the basis of discussions with the patient advisory group and a review of the literature on acceptability of anti-VEGF injections for wet AMD, regarding the factors that would influence choice of treatment. In selecting the attributes, our focus was on factors that would change across the three treatment regimens, based on assumptions made at the time of study design (in autumn 2020) that more frequent injections of complement inhibitors would be more efficacious but with a higher risk of developing wet AMD. Indeed, these three main factors (frequency, efficacy, side effects) are central to DCE studies carried out in the context of wet AMD injections (16–18). Some additional factors, such as waiting time in clinic and the pain or discomfort associated with injections, are also known to be burdensome for patients undergoing regular intravitreal treatment for wet AMD (19). We included waiting time in the scenario options because this was an important concern for the patient advisors; however, this was held constant across options, on the basis that the actual injection procedure and time in clinic would be similar whatever the treatment regimen. Similarly, it was explained in the information for study participants that the injection process for any of the GA treatment regimens would be similar to the wet AMD injection procedure, carrying the same risk at each injection of (non-clinically significant) pain and discomfort. The literature also suggests that clinically non-significant pain and discomfort is not a significant factor in affecting wet AMD patients’ decision-making and adherence or persistence with treatment beyond the first injection [20, 21], despite the potentially long-lasting impact of difficult or painful treatment experiences [22].

Decisions around the exact structure and language of the option cards were made in consultation with the patient advisory group. For example, we had initially considered using visualisations to communicate the information, but our advisory group discouraged this due to accessibility concerns. Similarly, a previous iteration of the option cards asked the participant to pick an option for themselves, rather than for the imaginary patient ‘Mr Smith’. However, many of the advisors found it confusing to select a hypothetical option for themselves, if for example their vision loss was already too advanced for watching television. Indeed, watching television was recommended as the activity to stand in for the functional vision preservation attribute, rather than activities such as reading the newspaper or driving, which the advisory group considered potentially less inclusive.

Data from the forced-choice task were audio-recorded, transcribed verbatim, and analysed using the Framework Method [23, 24]. A deductive approach to analysis was employed, whereby the attributes of the different treatment options (displayed in Table 1) were used as thematic categories to organise participants’ responses within a framework matrix. The framework matrix subsequently facilitated analysis of the convergence and divergence between participants’ views on the specific treatment attributes. NVIVO V.10.2 software (QSR International, Cambridge, Massachusetts, USA) was used to manage the qualitative data.

## Results

Twenty-eight of the overall 30 study participants took part in the forced-choice exercise. Baseline demographics of participants are presented in Supplementary file 1. The quantitative results, in terms of choices made, are shown in Table 1. Participants were classified as a clear proponent of one option if they consistently selected that option each time it was presented. Participants were classified as “No clear preference” if their choices showed no consistent preference (e.g. if they rated Option 4 as preferable to Option 1, Option 1 as preferable to Option 3, but then Option 3 as preferable to Option 4).

There was no correlation between option preferences and demographic factors including age, visual acuity and intravitreal injection exposure.

Table 2 displays participants’ reasoning about specific attributes of the different treatment scenarios. As shown, there was significant heterogeneity in participants’ views, with opposing logics influencing their decision-making; this highlights the highly personal and idiosyncratic nature of treatment preferences and is a noteworthy result. For example, quotations (q) 1 and 2 in Table 2 demonstrate concerns around the short time by which treatment would extend participants’ visual function, particularly in light of already advanced age. In contrast, participants in q3-4 justify their choice on the basis that any benefit for visual function, however limited, would still be helpful. While participants generally expressed the view that more frequent injections were burdensome (q6-7), one participant was in favour of more frequent injections because it would allow for more regular monitoring (q5). Time spent in clinic was rarely discussed (q8), because this was consistently set at two hours in clinic across all treatment options (apart from Option 1, no treatment). Risk of wet AMD was a factor that encouraged several participants to opt for lower risk (but also less efficacious) options (q9-11), although other participants (q12-13) were less concerned given the existence of effective intravitreal injection treatments for wet AMD. Notably, this minimal concern about risk of wet AMD applied for participants naïve to intravitreal injections (as in q12-13), as well as for certain participants already being treated successfully for wet AMD, e.g. “*His eyesight has improved with those injections… I say it’s improved, it’s steadied it. So the injections are working… That influences the decision*.” (P16 – daughter speaking on behalf of father – O3).

**Table 2:** Participants’ reasoning regarding their treatment preferences, with example quotations

Attribute and levels	Reasoning guiding participant’s preference	Example quotation (q) Parentheses following the quotation refer to the participant number, and their overall preferred option(s)
Preserved vision for… ***O1***: *5 years (no treatment, baseline)* ***O2***: *6 years (20% slowing)* ***O3***: *6.5 years (30% slowing)* ***O4***: *5.5 years (10% slowing)*	Relatively small magnitude of efficacy	1. “*[There’s] not much difference between 5 years, 5 and a half, or 6 and a half. So I would choose no treatment or less injections.”* (P5 – O1 = O2) 2. “*If my fellow eye was affected, I would be more interested. I am 85 years old, if I have 2 more years I will be satisfied. So 1.5 years is not long enough. If you said 10 years it would be different*.” (P10 – O4)
	Any preservation of visual function is beneficial	3. “*No injection option is out of question… Even if six months [more vision] is not long, it is still better. I know there’s a risk of wet [AMD], but longer vision is better*.” (P29 – O3) 4. “*I know six months is precious. But it’s not very long. I’d still go for Option 3… Yes, I’d try to get the maximum benefit*.” (P26 – O3)
Frequency of hospital visit and injection: ***O1***: *None (no treatment, baseline)* ***O2***: *6 times per year (once every two months)* ***O3***: *12 times per year (monthly)* ***O4***: *4 times per year (every three months)*	Frequent injections are preferable	5. *“I would like to have more frequent injections, ideally four weekly [ie once a month, Option 3], because I feel safer under close monitoring.”* (P8 – O3)
	Frequent injections are burdensome	6. “*Twelve injections that’s a lot. But it depends. Less injections is better. Twelve is a lot - I might even forget*.” (P23 – O4) 7. “*If I was to sell this to my mother… and the amount of effort she would have to make to come to hospital. And given her life expectancy. She would go for minimum injections. Because of my mum’s age and health*.” (P25 – son speaking on behalf of mother – O1)
Time spent at clinic: ***O1***: *None* ***O2***: *Up to two hours* ***O3***: *Up to two hours* ***O4***: *Up to two hours*	Time at the eye clinic is burdensome	8. “*Two hours is a long time. Not many people will be happy.”* (P23 – O4)
Risk of developing neovascular AMD within a year of starting treatment ***O1***: *1 in 50 (no treatment, baseline)* ***O2***: *1 in 20* ***O3***: *1 in 10* ***O4***: *1 in 50*	Risk of wet AMD as drawback to more frequent, effective treatment	9. *“It [concern about wet AMD] is my biggest thing. Because I’ve been told that’s worse than the dry one. They’re trying to stop the dry one developing into the wet one… the one in 50 chance of not getting it [in Option 4], that’s what sways me.”* (P13 – O4) 10. [Discussing Option 3] *“One in 10 chances of AMD, then I will have to have injection for wet. I think it’s not an option. I still don’t know.”* (P28 – O2) 11. [Discussing Option 2] “*The risk of wet AMD is off-putting as well. If you are going to get it anyway, like 1 in 20.*” (P25 – O1)
	Risk of wet AMD not affecting decision-making	12. “*I’m not concerned about the risk of wet AMD in particular*.” (P7 – O2 = O3) 13. [Discussing Option 3] “*He has a one in 10 chance of developing wet AMD. That’s interesting, isn’t it? That’s quite high, one in 10. So I have to make the same assumption that that [wet AMD] isn’t a disaster. Because it’s quite likely”.* (P26 – O3)

While Table 2 parses participants’ comments by attribute, more commonly participants discussed the different attributes holistically, in relation to each other rather than in isolation. For example, participants frequently contrasted increased preservation of visual function against the increased risk of wet AMD, e.g.:

“*It’s too small an increment. I would take less risk. The maximum gain is 6 months and the risk doubles. The gain isn’t worth the disadvantage.*” (P28 – O2 – explaining choice of O2 over O3).

A similar process was evident in discussing preservation of visual function versus the frequency of injections:

“*Only half a year [more vision] if I go twelve times against six times. I’d rather go six times, cos half a year’s not gonna make much difference… in that respect. If it was two years difference, then I would think about it*.” (P9 – O4 – explaining choice of O2 over O3).

“*It depends on the position of the eye, the situation of the eye, I can’t say. Because one year is certainly very useful, but I’m already 87… Option 3 is pathetic, it’s only half a year more than Option 2*.” (P30 – O2).

Despite the task involving a choice for the imaginary patient “Mr Smith”, most participants clearly related the decision-making process back to the particularities of their own situation; this is notable. For example, one participant stated:

“*If injections could guarantee it will improve my vision, I would go for injections. I chose no injection options because I am old enough. If I carry on for another four years, I don’t care what happens after that. For younger people it’s different. They are the ones to get old and blind. If they are young, for them it’s better than me. I am old enough. If I lose my eye it’s too bad.*” (P22 – O1).

This quote demonstrates that the participant understood the five years of preserved vision for Mr Smith in Option 1 as directly relevant to her situation, stating that she would be happy with even four years of preserved vision. However, she was able to put herself in the shoes of a younger person with GA who may see the treatments as more worthwhile.

Relatedly, several other participants who were overall ambivalent or negative about treatment struggled to make a choice. For example, P3 stated,

“*None of the options with treatment is acceptable for me and I am just answering, not that I would go for it. I really don’t see any benefits.*” (P3).

As noted above, six participants made choices in such a way that no clear treatment preference emerged. Conflicting pressures and priorities may provide one possible explanation for this; for example, P14 opted for each treatment option (O2, O3 and O4) twice. Explaining their decision-making, P14 stated, “*Risk of injections is a worry, but we know most things have risks. But I still think I would go if it’s not too many visits. If I have it six times a year, it’s a very small difference in years*.” This quotation demonstrates that the participant, while equivocal about the risks associated with treatment, is particularly torn regarding the balance between the frequency of visits and the relatively small magnitude of treatment efficacy, even with the every-other-month regimen (O2).

## Discussion and limitations

This forced-choice exercise using DCE-style treatment scenarios demonstrated that participants were generally, although not unanimously, in favour of less frequent treatment and lower risk of wet AMD, with Option 4 (one injection every three months, 1 in 50 risk of wet AMD) as the most popular option overall. This preference should be considered in line with 24-month results from Phase 3 clinical trials of pegcetacoplan, demonstrating minimal differences in GA growth reduction when treatment was given every month versus every-other-month [7]. Conversely, monthly injections in these trials were associated with a near doubling of the rate of exudative choroidal neovascularisation (12.2% in monthly versus 6.7% when treated every other month over 24 months).

We set out to translate treatment benefits to patients with GA in a meaningful way. Our study’s patient advisors agreed that time to central visual loss better conveyed the potential benefit of treatment, in comparison to percentage slowing of progression as reported in trial data. However, whilst trial data demonstrates statistically significant slowing of geographic atrophy lesion growth, which can be expected to result in preserved visual function for longer, none of the trials has so far demonstrated pre-specified functional benefit. This may be due to the heterogeneity of the trial participants and/or the current lack of structural endpoints that correlate well with visual function in GA [25].

Participants in the task were able to weigh the trade-off between more time with preserved central vision, frequency of clinic visits for injection treatments and risk of wet AMD. Whilst there were heterogeneous views and nuances around decision-making based on personal patient characteristics, overall this study suggests that efficacy, frequency of injections and risk of wet AMD are important attributes that will drive patient acceptability of these treatments as they become licensed.

This study had a number of limitations. Firstly, participants struggled with the exercise and did not always find it user-friendly, aligning with evidence regarding the challenge of ensuring participant understanding in more formal DCEs [26]. For example, our patient advisory group suggested DCE-style scenarios based on a hypothetical patient, yet many of the study participants interpreted the scenarios as having implications for their own clinical care. Secondly, some participants struggled with the notion of time remaining to watch TV, focusing on the specific activity itself rather than seeing it as an example for how long functional vision would be preserved. For instance, one participant stated: “*Television doesn’t sway me in the slightest so I don’t care if I could watch it for another ten years*” (P3). Thirdly, the forced-choice task was conducted at the end of a long interview, and two participants declined to participate. Fourthly, because the task was conceived as a prompt to elicit qualitative data rather than a more formal, quantitative DCE, the study did not allow us to consider how strongly one treatment (or specific level of an attribute) was preferred over another. Future work in this vein could use a measure to document the ‘weight’ of participants’ preferences, in order to encapsulate their sense of confidence or conviction in their choice, and thereby illustrate the extent to which one treatment option is preferred over another. Fifthly, in the case of three participants, an accompanying relative/caregiver helped to interpret parts of the interview; this was a means of involving a diverse participant cohort, representative of the multilingual population in the community. However, these proxy responses may not accurately reflect the participants’ actual preferences and decision-making processes, thereby potentially introducing bias into the study. Finally, attributes of the treatments were based on the Phase 2 trial data, available in 2020 when the study was designed. Both the treatment efficacy and risk of developing wet AMD were lower in the Phase 3 than Phase 2 studies.

Despite these limitations, overall we saw this forced-choice exercise as a useful tool for eliciting qualitative data, rather than a robust measure of treatment preferences. The importance of the attributes identified as drivers of acceptability in this exercise will be explored further in a larger UK-wide quantitative study.

### Electronic supplementary material

Below is the link to the electronic supplementary material.

Supplementary Material 1

## Data Availability

The full qualitative datasets generated during and analyzed during the current study are not publicly available, because the in-depth and specific information they contain could compromise the privacy of the participant. However, segments of qualitative data relating specifically to the forced-choice exercise, as well as the disaggregated quantitative data, are available from the corresponding author on reasonable request.

## Abbreviation

AMD: Age-related Macular Degeneration
DCE: Discrete Choice Experiment
GA: Geographic Atrophy
NHS: UK’s National Health Service
VEGF: Vascular Endothelial Growth Factor
