# Supplementary material for: Piloting a forced-choice task to elicit treatment preferences in geographic atrophy
Source: BMC Res Notes. 2023 Sep 30;16:244. doi: 10.1186/s13104-023-06531-8 (PMC10543887; doi:10.1186/s13104-023-06531-8)
Supplement: Supplementary file 1 — Supplementary Material 1 [file 13104_2023_6531_MOESM1_ESM.docx]

# Supplementary file 1: Demographic and clinical characteristics of study participants

| **Participant ID** | **Age** | **EQ-5D score** | **History of intravitreal injections? (Y/N)** | **Centre involving GA? (Y/N)** | **Better eye VA** |
| --- | --- | --- | --- | --- | --- |
| P1 | 83 | 41312 | Y | N | 0.24 |
| P2 | 87 | 21432 | Y | Y | 1.08 |
| P3 | 84 | 21213 | Y | N | 0.38 |
| P4 | 79 | 11132 | N | Y | 0.2 |
| P5 | 77 | 11111 | Y | N | 0.04 |
| P6 | 69 | 45511 | Y | N | 0.22 |
| P7 | 91 | 32211 | N | N | 0.34 |
| P8 | 80 | 21111 | Y | N | 0.4 |
| P9 | 77 | 11121 | N | N | 0 |
| P10 | 90 | 31223 | Y | N | 0.36 |
| P11 | 85 | 11111 | N | N | 0.16 |
| P12 | 75 | 11111 | N | Y | 0.06 |
| P13 | 75 | 51231 | Y | N | -0.1 |
| P14 | 82 | 33314 | N | N | 0.32 |
| P15 | 82 | 11111 | Y | N | 0.3 |
| P16 | 86 | 21131 | Y | N | 0.12 |
| P17 | 91 | 31313 | Y | Y | 0.5 |
| P18 | 76 | 11111 | N | N | 0.22 |
| P19 | 92 | 21311 | Y | N | 1.06 |
| P20 | 93 | 44411 | N | N | 0.22 |
| P21 | 70 | 11111 | Y | Y | 0.7 |
| P22 | 87 | 11311 | Y | Y | 0.6 |
| P23 | 87 | 31333 | Y | Y | 0.92 |
| P24 | 85 | 21111 | Y | N | 0.02 |
| P25 | 82 | 34443 | Y | N | 0.26 |
| P26 | 78 | 32222 | Y | Y | 0.6 |
| P27 | 79 | 11112 | N | N | 0 |
| P28 | 82 | 31111 | Y | Y | 1.06 |
| P29 | 84 | 32232 | N | Y | 0.3 |
| P30 | 87 | 44441 | N | Y | 0.7 |
